# Transepithelial Photorefractive Keratectomy—Review

**DOI:** 10.3390/vision8010016

**Published:** 2024-03-21

**Authors:** Christopher Way, Mohamed Gamal Elghobaier, Mayank A. Nanavaty

**Affiliations:** 1 University Hospitals Sussex NHS Foundation Trust, Eastern Road, Brighton BN2 5BF, UK; christopher.way@nhs.net (C.W.); mohamed.ebidalla1@nhs.net (M.G.E.); 2 Brighton & Sussex Medical School, University of Sussex, Falmer, Brighton BN1 9PX, UK

**Keywords:** photorefractive keratectomy, transepithelial, PRK, transPRK, excimer laser

## Abstract

The type and nature of refractive surgery procedures has greatly increased over the past few decades, allowing for almost all patient populations to be treated to extremely high satisfaction. Conventional photorefractive keratectomy involves the removal of the corneal epithelium through mechanical debridement or dilute alcohol instillation. An improvement to this method utilises laser epithelial removal in a single-step process termed transepithelial photorefractive keratectomy (transPRK). We explore the history of transPRK from its early adoption as a two-step process, identify different transPRK platforms from major manufacturers, and describe the role of transPRK in the refractive surgery armamentarium. This is a narrative review of the literature. This review finds that TransPRK is a safe and effective procedure that works across a variety of patient populations. Though often not seen as a primary treatment option when compared to other corneal-based procedures that offer a faster and more comfortable recovery, there are many scenarios in which these procedures are not possible. These include, but are not limited to, cases of corneal instability, previous refractive surgery, or transplant where higher-order aberrations can impair vision in a manner not amenable to spectacle or contact lens correction. We discuss refinements to the procedure that would help improve outcomes, including optimising patient discomfort after surgery as well as reducing corneal haze and refractive regression.

## 1. Introduction

Excimer and femtosecond laser systems have been deployed in a number of refractive procedures, including photorefractive keratectomy (PRK), laser in situ keratomileusis (LASIK), and intrastromal lenticular extraction. Conventional PRK involves the removal of the corneal epithelium through mechanical debridement or dilute alcohol instillation, or with a rotating brush. This can result in asymmetric stromal hydration and inaccurate ablation, delayed wound healing, pain, and a slower visual recovery [1]. This is relatively imprecise, and the resulting epithelial removal has an irregular edge and is larger than is required for the exposure of target stroma [2]. An improvement to this method involves removing the epithelium with an excimer laser with extreme precision, followed by a refractive stromal ablation in a single-step, no-touch method termed transepithelial PRK (transPRK). This avoids some of the issues of conventional PRK and includes a shorter operative time, decreased postoperative discomfort, and a faster visual recovery. It also avoids the creation of a flap, as seen in LASIK, meaning there are no flap-related complications.

## 2. History

TransPRK has been considered since the 1990s [3], where it was shown to have good visual outcomes compared to mechanical epithelial debridement [4,5,6]. However, early iterations were not effective by modern standards for a number of reasons:

1. They included separate laser modes through a phototherapeutic keratectomy (PTK) mode for epithelial removal, which switched to a PRK mode for the refractive stromal cut. The switch between modes was time-consuming, sometimes requiring a change in laser systems, resulting in unpredictable stromal dehydration and irregular ablation surfaces.

2. The PTK mode assumed a uniform epithelial thickness profile, which is now known to be false.

3. The flying-spot laser systems at the time created increased thermal effects and plume production centrally.

4. The peripheral cornea received less energy transfer owing to the increased distance from the laser and the oblique incidence of the light source on the periphery.

These issues resulted in issues of accuracy and predictability; earlier studies of transPRK found a slight overcorrection in refractive ablation, resulting in the addition of a +0.75D hyperopic modification into the laser settings [4,6,7]. However, even with normogram adjustments, it proved difficult to establish a refractively neutral epithelial ablation. Contemporaneous advancements in LASIK, a procedure with fast, accurate results, comfortable and minimal postoperative issues, and little to no corneal haze, meant it overtook PRK as the dominant refractive surgery procedure.

Over time, new laser platforms have addressed these issues: for example, the Nidek CXIII excimer laser adopted the Flex Scan algorithm to deliver greater ablations to the periphery. Modern systems ablate the epithelium and stroma in a single profile, minimising issues of asymmetric hydration between steps. We will discuss recent advancements in transPRK by identifying its role in the refractive surgeon’s armamentarium and discuss the platforms available and updates in strategies to minimise the issues surrounding transPRK that limit its more widespread use, namely postoperative pain, epithelial remodelling, and corneal haze.

## 3. Indications

The indications for transPRK are like that of conventional PRK and can be found in Table 1. It can be used as a treatment for both moderate [8,9,10] and high levels of myopia [11,12,13]. Data on hyperopia are limited by comparison [11,12,13], wherein there is more regression compared with myopia. Astigmatism is now treated in combination with spherical components [8,14]. Most surgeons recommend a residual stromal bed of between 250–320 μm and a Percent Tissue Altered (PTA) of <40% (though the majority of work on ectasia risk has been with LASIK, which confers a higher risk of post-surgical ectasia) [15,16,17,18].

LASIK and lenticular extraction are often used as the preferred primary treatment options, but both PRK and transPRK can often be used in situations where other procedures are contraindicated or to treat complications following other procedures. For example, LASIK and lenticular extraction are contraindicated in corneas with a thin planned residual stromal bed after flap creation; dry corneas, whereby flap creation will worsen dry-eye sensations; and cases when flap creation is considered risky based on patient factors such as professional participation in contact sports [21]. The lack of flap creation in transPRK allows this to be a viable treatment option in these cases. In transPRK, sub-Bowman stroma is not sacrificed by a cap or flap and can be utilised in the refractive ablation (preserving between 25 and 75 μm). This, combined with the lack of a flap- or cap-based limitation on the optical zone size, allows for large optical zones in low myopia (even exceeding 7.8 mm), which allows for high resulting visual quality [22,23].

Contraindications to transPRK include patient factors whereby healing is impaired, such as autoimmune diseases like rheumatoid arthritis and metabolic conditions like poorly controlled diabetes. Ocular conditions include any active ocular inflammation or infection and a past history of herpes keratitis (owing to a risk of reactivation). Corneal factors include low corneal thickness; deep corneal stromal scarring or vascularisation; unstable refraction, such as in young patients and in progressive myopic patients; hormonal instability, such as in pregnancy and breastfeeding, due to associated refractive instability; tomographic evidence of corneal ectatic conditions such as keratoconus; as well as uncontrolled glaucoma and steroid responders because of the requirement for long-term topical steroid use to prevent corneal haze. Patients with unrealistic expectations, particularly those who expect fast visual recovery after the procedure, must be counselled extensively if the procedure is to be recommended.

PRK ablation of the corneal epithelium can be utilised in a therapeutic manner (akin to a phototherapeutic keratectomy (PTK)). This includes epithelial pathologies such as anterior basement dystrophy or the removal of an anterior stromal scar caused by a contact lens-related ulcer. TransPRK is often a useful treatment option for patients who have had LASIK complications, including flap/interface complications and epithelial ingrowth [24,25,26].

Treatment of lower-order refractive errors can cause higher-order aberrations (HOAs) that reduce quality of vision [27]. HOAs such as coma, trefoil, and spherical aberrations must be considered, particularly in large refractive ablations [11], and an increase in trefoil aberrations can be seen in cases of astigmatic corrections [28].

## 4. Preoperative Epithelial Thickness Mapping

The corneal epithelium is around 50–52 μm thick centrally [29] and is thicker in the periphery [30]. It plays an important role in the net refractive power of the cornea, with a central power of 1.03 D in the central 2 mm and the power decreasing peripherally [31]. Using high-frequency ultrasound, Reinstein et al. produced a corneal epithelial thickness map showing thicker nasal and inferior portions with the location of the thinnest epithelium situated superotemporally from the corneal vertex [32]. Using spectral-domain OCT, Kanellopoulos and Asimellis similarly found the mean epithelial thickness to be thinner superiorly (51.86 ± 3.78 μm) than inferiorly (53.81 ± 3.44 μm) [33]. Both also similarly found high inter-individual variability in the epithelial thickness map. The thickness profile may be affected by factors such as age (either causing thinning [34] or thickening [35]). Patients with thinner epithelial thickness profiles will have excess stroma removed, and those with a thicker epithelial thickness will have a portion of their refractive cut that will only remove the epithelium, resulting in a smaller optical zone. Even if this is accounted for, the assumption of a uniform epithelial thickness through the cornea during ablation will result in epithelial breakthrough in thinner areas (mostly superotemporal) earliest and lead to asymmetric ablation profiles, inaccuracy, tilt, and coma in a manner that would be avoided with manual epithelial removal.

The variability of the epithelial thickness map means that the application of uniform epithelial settings to virgin eyes will lead to asymmetric and sometimes unpredictable epithelial breakthrough in transPRK. This is further complicated by some other issues:

1. The epithelium has a different refractive index compared to the stroma (1.401 vs. 1.377 [36]).

2. Though the epithelium compensates by thickening over areas of increased stromal removal [37,38], it is likely insufficient in its compensation.

3. The epithelium and stroma have differing rates of ablation [39]. However, this is minimised by the optimised energy fluence, shot pattern, and frequency of modern lasers.

Theoretical work from Arba and Awwad has suggested that a patient epithelium that is thicker than predicted would also result in a reduced optical zone, whereas a thinner epithelium would result in more stromal ablation than planned, particularly in the periphery [40]. Either way, an unpredictable epithelial lenticule breakthrough will impair the overall refraction accuracy. These effects are magnified in cases of small ablation and have resulted in an initial recommendation stating that transPRK should not be used in low myopia below −1.00D. To mitigate this, de Ortueta et al. increased the epithelial ablation profile and optical zone in low myopes to add an ablation buffer whilst keeping the refractive profile the same. The efficacy, safety, and predictability of this was equivalent when compared with moderate myopia [41].

To mitigate these issues, transPRK platforms plan to ablate 55 μm central epithelium and 65 μm epithelium in the periphery [10].

## 5. TransPRK Platforms

The Schwind Amaris system (Schwind eye-tech-solutions, Kleinostheim, Germany) combines PTK and PRK into a single-step reverse aspheric PRK to generate the refractive cut. This is based on a predictive estimation of epithelial thickness of 55 μm centrally and 65 μm peripherally, but this can be adjusted according to surgeon preferences [10]. The “Smart Pulse” ablation software (Schwind eye-tech-solutions, Kleinostheim, Germany) uses different ablative spot geometrics to reduce the thermal load and reduce ablation bed irregularities [42,43]. This was found to result in reduced recovery times, reduced pain in the first few days, and reduced haze in a retrospective non-blinded analysis [43]. This treatment can be used in aberration-free eyes (i.e., virgin eyes, as opposed to retreatment cases) as well as corneal WFG and ocular WFG ablations. The epithelial ablation profile assumes a gradual increase in epithelial thickness from the central to peripheral areas of the ablation zone and is slightly greater than the normal epithelium, in order to reduce the risk of incomplete epithelial removal and minimise any consequences relating to variability in the patient epithelial thickness map. The positive outcomes and clinical experiences on this platform are described by Ortueta et al. [44]. There is no interruption between epithelial and stromal ablations, which reduces treatment time. The Intelligent Thermal Effect Control (ITEC) software (Schwind eye-tech-solutions, Kleinostheim, Germany) limits the local frequency to 39 Hz while maintaining system repetition rates of 500/750/1050 Hz [45,46]. This spatial and temporal separation of laser pulses minimises the thermal load by allowing time for localised cooling. When tested with the Amaris 750 Hz excimer laser during transPRK, De Ortueta found that this kept the corneal epithelium below the 40deg C safety net thought to be the level at which collagen proteins start to denature [47,48]. Incremental improvements to these treatment normograms have shown improved outcomes with respect to HOAs [49].

The Alcon Streamlight (Alcon, Fort Worth, TX, USA) is another single-step transPRK platform, added to the EX500 Excimer laser platform in 2019. It utilises a set of algorithms (EPI lists) to provide a uniform PTK ablation of the epithelium in 5 µm steps to levels of a normal corneal epithelium (45–65 µm) after epithelial mapping and according to surgeon preference [50]. This PTK seeks to be refractively neutral. Wavefront-optimised (WFO) stromal ablations immediately follow the PTK procedure and utilise the same centration, size, and location of the PTK treatment zone. Like the Schwind software, there is no interruption between epithelial and stromal ablations, which reduces the treatment time. Using this platform appears to be a safe, effective treatment for low to moderate myopia and hyperopia with and without astigmatism, with the benefit of faster visual recovery and epithelial healing as well as less pain than traditional PRK [20,50,51]. Re-epithelialisation in particular was very fast when compared with alcohol-assisted PRK.

The iVis laser suite (iVisTechnologies, Taranto, Italy) combines its iRes excimer laser with the cTen™ platform to provide a single-step transepithelial PRK method. It utilises the planning software Corneal Interactive Programmed Topographic Ablation (CIPTA^®^), which recommends an ablation that intersects the patient’s topographic data and an aberration-free ideal shape, resulting in less tissue ablation in comparison to wavefront-guided ablation [52]. The excimer laser ablates up to 1000 Hz, but is adjusted to 5 Hz/mm^2^ to avoid thermal damage [52].

## 6. Comparisons with Other Refractive Surgery Options

Comparisons with the other refractive procedures, such as LASIK and lenticular extraction, can be found in Table 2.

## 7. Conventional PRK

Earlier iterations of the transPRK platform, using a PTK mode and with the difficulties described previously, resulted in inferior outcomes when compared to manual removal of the epithelium in domains including accuracy [5,6] (when using the Visx S3 laser, Johnson & Johnson, Irvine, CA, USA). Modern laser systems exhibit improved transPRK outcomes.

A meta-analysis by Alasbali et al. compared visual and patient-reported outcomes in studies comparing transepithelial PRK with conventional PRK [68]. It found that the efficacy and safety was equivalent, and the accuracy, as measured using the postoperative spherical equivalent, was slightly better in transPRK. Secondary outcome measure reporting was variable. TransPRK provided faster epithelial times and less pain compared with conventional PRK, whereas the incidence of postoperative haze was equivalent. Only two studies in the meta-analysis investigated patient satisfaction, with both showing higher satisfaction with transPRK [2,69], less postoperative pain [8,70], and faster re-epithelialisation and visual recovery [8,10,70,71], as well as shorter surgical times [2,69]. The reduced pain is probably multifactorial and is probably related to a faster re-epithelialisation for two reasons:

1. A smaller area of epithelium is removed in transPRK;

2. The lack of the temporary toxicity of alcohol on limbal stem cells that could be seen in alcohol-assisted PRK [72].

The longest follow-up in the meta-analysis was 40 months in a fellow eye-related study between alcohol-assisted PRK and transPRK from Rodriguez et al. [73]. They found no statistically significant difference in primary or secondary outcomes [73].

## 8. LASIK

Laser-in situ keratomileusis (LASIK) is the most widely adopted corneal refractive surgery option worldwide owing to its faster recovery, less pain, and less corneal haze than traditional PRK [57,58]. Earlier methods of flap generation were performed with a microkeratome. The advances in transPRK using the Nidek EC-500 (Nidek Inc., San Jose, CA, USA) excimer laser found slightly better visual outcomes than LASIK or laser-assisted sub-epithelial keratomileusis (LASEK) in both low and high myopia [74]. Modern flap creation uses a femtosecond laser and promises enhanced safety through accuracy and repeatability [75]. Gershoni et al. found slightly better outcomes of femtosecond LASIK compared to transPRK in low to moderate myopia [76], whereas Aslanaides found similar refractive outcomes and marginally favourable visual acuity outcomes to LASIK and PRK [10]. Ghadfan found improved visual outcomes with transPRK than LASIK, LASEK, and mechanical PRK [74], and when comparing transPRK with femtosecond LASIK, Zhang found equivalent safety and efficacy but fewer total higher-order aberrations in transPRK, owing to a higher induction of vertical coma with femtosecond LASIK [13,19].

## 9. Lenticule Extraction

Lenticule extraction procedures are offered on several platforms. Small-incision lenticule extraction (SMILE) is the most published lenticular extraction procedure. Lenticular extraction offers benefits over LASIK in the preservation of the biomechanically important anterior stroma, allowing the use of larger optical zones and maintaining the anterior corneal nerve plexus important in corneal afferent pathways [77,78,79,80]. When compared with conventional lenticular extraction, wavefront-guided transPRK was similarly effective and safe, but the total HOAs was greater in transPRK even on wavefront-guided terms [81]

## 10. Biomechanical Stability Advantages

Corneal laser refractive surgery induces a degree of corneal biomechanical instability. The interest in this area is born from the incidence of post-laser-vision-correction corneal ectasia [82,83,84]. Though the known risk factors for the development of post-surgical ectasia include a low residual stromal bed and a high percentage of tissue altered, idiopathic cases of ectasia still develop, often 2–8 years after the refractive procedure [61,85,86]. Xin et al. compared the difference in corneal stiffness between transPRK, lenticular extraction, and FS-LASIK. All patients undergoing procedures exhibited reduced corneal stiffness postoperatively, but the reduction was least in those undergoing transPRK [63].

## 11. Use in Complex Corneas

TransPRK is useful in corneas that have been affected by previous surgery or insult such as keratoplasty and radial keratotomy. These cases present an interesting challenge owing to the high levels of ametropia and astigmatism, impaired epithelial behaviour, and interaction of an underlying disease process. Spectacles are often insufficient in these cases as the refractive error is not simply spherocylindrical, and scleral or rigid gas-permeable contact lenses are often not tolerated and come with their own complications [87,88].

Post-keratoplasty astigmatism treated with LASIK is effective but often causes refractive regression and corneal haze. Post-keratoplasty eyes subjected to LASIK are at risk of perforation and endothelial cell loss owing to different flap generation and manipulation [89,90,91]. Complications in a series by Spadea et al. of 12 PKP patients undergoing LASIK included the formation of corneal haze in 2 and graft rejection in 1, which was managed with topical steroids [92]. The follow-up was short when one considers the fact that the main issues of regression and corneal haze can develop many years postoperatively. Radial keratotomy is also not best served with the creation of a flap or lenticule, as uncontrolled shearing forces can cause flap-related complications, perforation, or extension of the keratotomy wound lines and epithelial ingrowth [93]. Though it is difficult to generate large numbers of these heterogenous groups of patients, both wavefront-guided and topography-guided transPRK in post-keratoplasty and post-RK patients appear safe and effective [92,94,95].

Keratoconus presents an interesting challenge to the surgeon as the risk of inducing further ectasia is among the most feared complications of corneal refractive surgery. However, the higher-order aberrations (HOAs) associated with keratoconus progression are visually disabling and are not corrected well with spherocylindrical spectacles or contact lenses. Treatment of HOAs with transPRK ablation of corneal stroma improves visual quality. Simultaneous CXL/transPRK has been shown to be safe and not weaken the effect of CXL in patients with mild to moderate keratoconus [96,97]. To limit the biomechanical effects of stromal ablation, less tissue and a smaller ablation depth is used (50 μm, as recommended by Kanellopoulos) [96]. Usually, when aiming to treat HOAs, the lower-order aberration target is modified to zero (i.e., no refractive ablation). However, this results in further tissue ablation than necessary as ablation of the HOAs results in spherocylindrical modification, and further tissue is excised to restore this to refractive neutrality. Platforms such as the Schwind Amaris system allows for decoupling of these targets, and when Gore et al. treated HOAs only and ignored the influence on lower-order aberrations, 30% less tissue was removed. Visual quality was improved compared with CXL alone and corneal stability was maintained for a 24-month follow-up [97]. The remaining spherocylindrical component was treated with other methods such as spectacles or phakic intraocular lenses [94].

## 12. Wavefront-Guided Aberrations

Like all other corneal refractive surgery options, transPRK treatments result in an increase in higher-order aberrations. This is particularly important in hyperopic, high-volume, and high-astigmatic ablations. A vector analysis by Jun et al. suggested that in moderate to high astigmatism, corneal wavefront-guided (CWFG) transPRK results in a more predictable astigmatism correction as well as fewer higher-order aberrations, particularly coma, than WFO transPRK [98]. High myopic astigmatism presents issues with asymmetric ablations and the induction of HOAs. A corneal wavefront-guided transPRK induced fewer aberrations than either an aberration-free profile or a wavefront-optimised profile [98,99].

TransPRK was associated with a higher amount of HOAs than alcohol-assisted PRK [49]. Lee et al. found higher amounts of HOAs with transPRK than with conventional lenticular extraction, even when the transPRK was performed under WFG conditions [81]. This is perhaps due to the asymmetric epithelial breakthrough discussed previously. For patients with pre-existing high levels of HOAs, WFG transPRK treatments were not found to significantly increase the HOAs when compared with aberration-free treatments [100].

## 13. Issues

The disruption to the integrity of the epithelium and anterior stroma from surgery causes keratocyte migration, and the deposition of glycosaminoglycans and collagen into the anterior stroma during the healing phase. If this causes clinically significant opacification, it is known as corneal haze; this can reduce the quality of vision significantly. Several factors are thought to increase the likelihood of haze formation. Primary risks including high tissue ablation and the laser energy settings, including thermal load. Secondary risks include the presence of dry eye disease, diseases that compromise wound healing, including autoimmune diseases, as well as postoperative UV exposure [101,102,103,104,105,106].

Topical use of off-label mitomycin-C (MMC) is commonly used at the end of treatment to reduce haze through its mechanism of myofibroblast inhibition and reduction in keratocyte activity [107,108]. However, the widespread activity creates concerns over its effect on other ocular structures such as the limbus, tear film, or corneal endothelium. A meta-analysis by Oerdane et al. suggested that postoperative MMC reduces the risk of haze formation at 6 and 12 months (*p* < 0.00001) and may slightly improve overall visual acuity at 5 years compared with controls (*p* = 0.05) [109]. There was no significant impact of MMC on spherical equivalent, endothelial cell loss, or other side effects [109]. However, the long-term effects of MMC are not established and for this reason it is not universally used in the refractive surgery community.

The incidence of thermally induced haze can be reduced with several methods, including the optimised ablation platforms that reduce the thermal load and leave behind a smoother stromal surface. Intraoperative routine usage of chilled balanced salt solutions and a time gap between epithelial and stromal ablation helps to limit the maximum temperature below the safe threshold of 40 °C, as recommended by De Ortueta [47], though this does increase the treatment time. Managing the thermal load will also decrease ocular surface pain after the procedure [110]. Abdelwahab et al. found no significant haze in their cohort of 500 eyes with low to moderate myopia treated with single-step transPRK using WaveLight™ (Alcon Laboratories, Fort Worth, TX, USA). For their study, 0.02% mitomycin C was applied for 20 s when the ablation depth was greater than 60 µm. Only 17% of eyes had grade 0.5 haze at 3–6 months (i.e., not clinically significant or symptomatic), and no patients had haze at the last follow-up visit [50].

Other topical anti-haze strategies include postoperative topical corticosteroids, which appear to reduce haze without impeding the re-epithelialisation time; however, the side effects of topical steroids are well known [111]. The antihypertensive agent losartan is known to inhibit TGF-β1 signalling and type IV collagen deposition. Its application on rabbit corneas subjected to a −9.00 D PRK reduced haze compared with controls [112]. Further investigation into this medication’s effect on transPRK patients is welcome [113].

Postoperative corneal pain is a significant limiting factor in the popularity of this procedure. Various intra- and postoperative factors can help control postoperative pain. Certainly, the reduced thermal load offered by modern treatment platforms leads to reduced pain [114]. The many postoperative regimens available include topical NSAID drugs, topical steroids, cycloplegics, and oral analgesics [115,116]. Postoperative oral gabapentin was found to be ineffective in controlling postoperative pain [117]. A topical bandage contact lens soaked in ketorolac 0.45% reduced postoperative pain more than a bandage contact lens alone after transPRK [118]. Topical anaesthetics have been well employed in limited dilute concentrations to avoid epithelial toxicity [119]. The authors are aware of high-dose oral steroids being used in the perioperative period in conventional PRK and transPRK with proponents claiming a faster visual recovery, less pain, and minimal side effects if the steroids are tapered quickly. We welcome published trials demonstrating this effect.

Vitamin C levels in the tear film reduce after transPRK [120]. However, oral supplementation did not improve haze, subjective pain, or re-epithelialisation time compared with a placebo [121]. Vitamin A is important in limbal stem cell differentiation and wound healing [122]. Vitamin E is an antioxidant that may reduce keratocyte apoptosis after PRK [123]. When combined together, vitamin A and E supplementation was associated with faster re-epithelialisation times and a slightly lower incidence of post-PRK haze [124]. Whether there is a true benefit to be found remains to be seen and further investigation is welcome in this area.

## 14. Towards the Future

Future developments in software algorithms may utilise individual epithelial thickness mapping to ensure even epithelial breakthrough and minimise redundant stromal ablation. Transepithelial ablations cause higher thermal loads than intrastromal ablations [124]. This should be expected; transPRK treatments require more overall ablation volumes than stromal ablation and the epithelium is more sensitive to laser energy, giving a higher response to ablation [47,125]. Future higher-frequency excimer systems must develop advanced thermal control software if transPRK is to be adopted with higher frequencies.

Advanced ablation profiles will help to control and utilise higher-order aberrations and allow us to enhance image quality. Significant postoperative haze has been largely reduced through the use of MMC, but this is not a universal adoption owing to concerns over long-term safety. Other medications with less anticipated toxicity are welcome. Practice patterns for the postoperative setting should be shared widely, but improvements to postoperative pain and faster re-epithelialisation require robust and well-powered clinical trials. Meta-analyses are welcome, as have been performed between transPRK and conventional PRK [68], but secondary outcome reporting is variable and standardisation in secondary reporting outcome measures for a refractive surgery procedure are welcome. One recognises that advancing technologies are difficult to subject to systematic review as they are subject to evolution. In addition, long-term data are difficult to achieve but are essential, as epithelial and stromal remodelling after PRK is known to occur many months after surgery [1]. Myopic regression is known to occur more commonly with traditional PRK than LASIK [126]. O’Brart found that the stability from traditional PRK at 1 year was maintained for up to 7.5 years [127]; logically, these behaviours should also apply with transPRK.

Despite the multiple comparison studies described above, many have not included patient reported outcome measures (PROMs). Given that the accuracy and safety profile of transPRK correction procedures is extremely high, improvements to the field are of marginal gain, which will require robust well-controlled prospective studies to improve outcomes.

## 15. Conclusions

Overall, transPRK is a safe, efficient, and effective part of the refractive surgery space. It occupies an important role in patients who may be ineligible for other forms of refractive surgery, such as those with thin corneas, high myopia, concomitant epithelial disease, and in cases of retreatment or early keratoconus. Pain, slow and irregular epithelial healing, and corneal haze limit its use as a primary treatment option, particularly within the context of the fast, effective, comfortable flap-based procedures of LASIK and intrastromal lenticular extraction. However, the pain and recovery associated with transPRK is superior compared with conventional PRK. The single-step nature of modern platforms allows for a fast, simple procedure that is suitable for high-volume practices.

## Figures and Tables

**Table 1:** Suggested maximum refractive error eligible for transPRK.

Correction	Maximum Dioptric Correction
Myopia	Sphere: −10.00 D [19]
Myopic Astigmatism	Sphere: −10.00 D [19] Cylinder: −6.00 D [14] Vector Sum of Sphere and Cylinder: <10.00 D [19]
Hyperopia	Sphere: +3.00 D [20]
Hyperopic Astigmatism	Sphere: +3.00 D Cylinder: +3.00 D [20]
Mixed Astigmatism	Sphere: −4.00 D [21] Cylinder: +6.00 D [21]

**Table 2:** Comparisons between the major methods of corneal refractive surgery. LASIK: laser in situ keratomileusis, PRK: photorefractive keratectomy.

Aspect	Lasik	Lenticule Extration	Conventional Prk	Transepithelial Prk
Surgical Procedure	Flap creation followed by laser ablation on the stromal bed	Intrastromal corneal lenticule excision	Epithelial removal with alcohol or rotating brush followed by laser ablation	No-touch surface ablation without flap
Flap Thickness	Deeper flap creation (typically around 90–120 µm) [53]	No flap creation. Involves a small incision (4 mm). The part of the stroma anterior to the lenticule is called the cap. Normal cap thickness varies from 110–140 µm [54]	No flap creation, surface ablation	No flap creation, surface ablation
Recovery Time	Rapid visual recovery (usually within 24 h) [55]	Quick recovery (often within a few days) [56]	Longer initial recovery (several days to weeks)	Longer initial recovery than LASIK or lenticule extraction, faster recovery than conventional PRK
Pain And Discomfort	Minimal discomfort after surgery [57,58]	Generally, less discomfort compared to LASIK [59]	Discomfort during recovery	More discomfort during recovery than LASIK or lenticule extraction, less discomfort than conventional PRK
Dry-Eye Symptoms	Potential for temporary dry eyes [60]	Lower risk of dry-eye symptoms compared to LASIK [60]	Less dry-eye symptoms than LASIK	Less dry-eye symptoms than LASIK
Suitability For Thin Corneas	Less suitable [61]	Less suitable	May be suitable [62]	May be suitable [63]
Enhancement Procedures	Easier to perform enhancements [64]	More challenging to perform enhancements [65]	Possible	Possible
Overall Vision Quality	Excellent [55]	Excellent [55]	Excellent but takes longer to stabilise [66]	Excellent but may take longer to stabilise [67]

## Data Availability

Not applicable.
